# Drinking Water Week — May 5–11, 2013

**Published:** 2013-05-03

The United States has one of the safest public drinking water supplies in the world (1). Tap water not only provides water for daily activities such as drinking, bathing, and cooking, it also benefits the entire community by providing water to serve businesses, schools, and hospitals, and to promote overall health (2). May 5–11, 2013, is Drinking Water Week, an annual observance whose theme “What Do You Know About H_2_O?” underscores the many ways in which all consumers can get to know their water (3).

Disinfection and treatment practices, as well as the environmental regulation of water pollutants, have substantially improved domestic water quality during the past century and have led to a marked decrease in the incidence of waterborne diseases such as typhoid fever (4–6). Despite these improvements, sources of drinking water still can become contaminated and lead to adverse health effects (7).

New challenges to the U.S. water supply include aging drinking water infrastructure, the impact of climate change on water availability and quality, chemical contamination of water sources, emerging pathogens, and the development of new ways to obtain and use water. Drinking Water Week is a time to highlight the importance of safe drinking water and recognize that protecting and reinvesting in water infrastructure is crucial to the health of persons living in the United States.
